# Rex Shunt Preoperative Imaging: Diagnostic Capability of Imaging Modalities

**DOI:** 10.1371/journal.pone.0022222

**Published:** 2011-07-12

**Authors:** Sharon W. Kwan, Nicholas Fidelman, Jeremy C. Durack, John P. Roberts, Robert K. Kerlan

**Affiliations:** 1 Division of Interventional Radiology, Department of Radiology and Biomedical Imaging, University of California San Francisco, San Francisco, California, United States of America; 2 Department of Surgery, University of California San Francisco, San Francisco, California, United States of America; Universität Würzburg, Germany

## Abstract

The purpose of this study was to evaluate the diagnostic capability of imaging modalities used for preoperative mesenteric-left portal bypass (“Rex shunt”) planning. Twenty patients with extrahepatic portal vein thrombosis underwent 57 preoperative planning abdominal imaging studies. Two readers retrospectively reviewed these studies for an ability to confidently determine left portal vein (PV) patency, superior mesenteric vein (SMV) patency, and intrahepatic left and right PV contiguity. In this study, computed tomographic arterial portography allowed for confident characterization of left PV patency, SMV patency and left and right PV continuity in 100% of the examinations. Single phase contrast-enhanced CT, multi-phase contrast-enhanced CT, multiphase contrast-enhanced MRI, and transarterial portography answered all key diagnostic questions in 33%, 30%, 0% and 8% of the examinations, respectively. In conclusion, of the variety of imaging modalities that have been employed for Rex shunt preoperative planning, computed tomographic arterial portography most reliably allows for assessment of left PV patency, SMV patency, and left and right PV contiguity in a single study.

## Introduction

Mesenteric to left portal vein bypass within the Rex recessus, also known as a Rex shunt, is currently the preferred treatment for extrahepatic portal vein thrombosis (EPVT) in the pediatric and adolescent population [1]–[3] and is gaining support as a surgical option in adults with symptomatic EPVT and preserved hepatic function [4]. An advantage of the Rex shunt over traditional shunts is that it reestablishes physiological hepatopetal flow, resulting in improved hepatic function and favorable neurological and developmental outcomes, particularly in children [5], [6].

Preoperative imaging must demonstrate two criteria for a patient to be considered for placement of a traditional Rex shunt: 1) patency of the intrahepatic left portal vein (PV) and 2) a suitable and patent superior mesenteric vein (SMV). An ideal candidate should also have patency and contiguity of the intrahepatic left and right PV, although this is not a strict requirement [7]. A wide variety of imaging modalities have been used as part of the preoperative evaluation of these patients. These include minimally invasive examinations such as contrast-enhanced computed tomography (CECT), contrast-enhanced magnetic resonance imaging (CEMR), and ultrasound with color Doppler. Invasive diagnostic procedures such as trans-splenic portography, transhepatic portography, transarterial portography, and computed tomographic arterial portography (CTAP) have also been employed. To our knowledge, no published study exists on the diagnostic capability of these examinations for this indication.

The purpose of this study was to evaluate the diagnostic capability, i.e. the ability to confidently make a diagnosis, of different imaging modalities used for preoperative Rex shunt planning.

## Methods

### Patient population

This study was compliant with the Health Insurance Portability and Accountability Act and conducted according to the principals expressed in the Declaration of Helsinki. The University of California, San Francisco Medical Center institution review board approval was obtained, with the requirement for informed consent was waived for this retrospective study.

All patients referred to our center in a 10 year period (November 1998–November 2008) for Rex shunt evaluation were identified through a database search of relevant International Classification of Diseases (ICD-9) billing codes. The study group was comprised of 20 patients (9 male, 11 female, mean age 26 years, age range 19 months to 65 years). Electronic medical records were reviewed. Data collected included demographics and operative reports, where relevant. All abdominal imaging studies performed within six months of referral for clinical evaluation for Rex shunt placement were retrieved. The type and number of imaging examinations performed was at the discretion of the referring liver surgeon. Unenhanced computed tomography or magnetic resonance imaging examinations were excluded. If a type of imaging study was performed more than once, only the most recent study was included.

### Imaging protocols

CECT examinations were performed using a 4- or 16-detector row scanner (LightSpeed LX/i or LightSpeed; GE Medical Systems, Milwaukee, WI). The abdomen was imaged from the dome of the diaphragm to the iliac crests. Contrast used was 150 mL (adult) or 1 mL/pound (pediatric) of iohexol (Omnipaque-350; GE Healthcare, Princeton, NJ), injected intravenously through a power injector at a rate of 4–5 mL/s. Multiphase CECT included contiguous noncontrast images with 5 mm collimation followed by late arterial images with a 20 second delay and 2.5 mm collimation and portal venous phase images with a 70 second delay and 2.5 mm collimation. Single phase CECT included portal venous phase images following a 70 second delay with 5 mm (adult) or 2.5 mm (pediatric) collimation.

CEMR examinations were performed with a phased-array surface coil in a 1.5-T imager (Signa, GE Medical Systems). Axial dynamic three dimensional fat-suppressed spoiled-gradient echo sequences (typical parameters: TR = minimum/TE = minimum, flip angle, 15°–20°, section thickness 4–6 mm with 50% overlap, field of view = 32–40 cm) were used for evaluation of the SMV and PV. Contrast used was 0.2 mL/kg gadodiamide (Omniscan; GE Healthcare) or gadopentetate dimeglumine (Magnevist; Bayer HealthCare Pharmaceuticals, Wayne, NJ). Contrast timing was based on peak enhancement of the aorta with a test bolus. Arterial, portal venous, equilibrium and delayed contrast phases were obtained with following scan delays of 0, 8, 25 and 300 seconds, respectively.

Ultrasound examinations were performed with an Acuson Sequoia 512 real time system (Siemens Medical Solutions, Erlangen, Germany). Images were captured as deemed appropriate by the sonographer, and all examinations included image captures in the expected locations of the main, left and right PV. Interrogation with color Doppler was performed when these structures were identifiable.

Trans-splenic portography was performed with placement of a 5 French sheath over a 17 gauge needle (LR sheathed needle, Cook Inc. Bloomington, IN) into the splenic parenchyma under ultrasound guidance. Following splenovenography, the needle tract was embolized with autologous blood clot and gelfoam pledgets.

Transhepatic portography was performed with placement of a 21 gauge needle into an intrahepatic PV under ultrasound guidance. If no retrograde flow into the contralateral PV system was appreciable, direct puncture of that system was subsequently performed, followed by portal venography.

Transarterial portography was performed with a 5 French Cobra-2 catheter (Angiodynamics, Queensbury, NY or Cook, Inc.) placed into the proximal superior mesenteric artery and proximal splenic artery via right common femoral artery puncture.

For all angiographic and venographic procedures, iohexol contrast was used, with injection rate and contrast volume determined by the attending interventional radiologist.

CTAP was performed with a 5 French Cobra 2 catheter inserted into the proximal SMA under fluoroscopic visualization from a common femoral artery puncture. The catheter was secured at the groin and the patient transported to the CT scanner. A 50∶50 solution of sterile saline and iohexol was injected at 3 mL/s for a volume of 90 mL. Imaging was performed on a 16-detector CT with 1.25 mm collimation after scan delays of 15, 30 and 60 seconds.

### Image review

Two readers retrospectively reviewed by consensus all studies on a picture archiving and communication system workstation (Impax; Agfa, Mortsel, Belgium). If a patient underwent multiple planning imaging examinations, they were reviewed in chronological order. Each imaging examination was assigned to one of the following categories with respect to 1) left PV patency: patent, occluded, or non-diagnostic (cannot confidently determine); 2) SMV patency: patent, occluded, non-diagnostic, or not applicable and 3) intrahepatic left and right PV contiguity: contiguous, non-contiguous, or non-diagnostic. SMV patency was defined as a segment of the SMV contiguous with the splenic-PV confluence measuring at least 3 cm in length and 1 cm in width which demonstrated contrast enhancement. These SMV parameters are generally recognized by liver surgeons as adequate for Rex shunt creation. Left PV patency was defined as contrast enhancement or color Doppler signal within the left PV starting within 1 cm of the main PV and extending peripherally through the expected course of the first order intrahepatic left PV. PV contiguity was defined as any visible connection with demonstrable flow (as evidenced by contrast enhancement or color Doppler signal) between a dominant PV within the left and right hepatic lobes. Evaluation of SMV patency was not applicable for ultrasound, trans-splenic portography, and transhepatic portography.

### Statistical methods

All data were entered into a spreadsheet (Microsoft Excel; Microsoft, Redmond, WA) for descriptive analysis. Statistical significance of the difference between imaging modalities was determined with the Fisher exact test, a nonparametric test for association with small sample sizes. This analysis was performed with a standard statistical software package (STATA SE version 10.0; StataCorp, College Station, TX).

## Results

Twenty patients with EPVT underwent 57 preoperative planning abdominal imaging studies. The median number of examinations per patient was 3.0(range, 1–5).

The technical success rate for transhepatic portography was 10/11 (90.9%); in one case, access to the portal venous system could not be gained with the 21 gauge needle. This study was considered non-diagnostic in the data analysis. A technical success rate of 100% was achieved with all other modalities performed in the interventional suite (trans-splenic portography, transarterial portography, CTAP). There were no procedure-related complications.

Table 1 summarizes the diagnostic capability, defined as the number of studies resulting in confident diagnosis of patency versus occlusion or contiguity versus non-contiguity divided by the number of studies which did not allow for confident diagnosis, for each modality. The ability to determine left PV patency varied widely. Single phase CECT, multi-phase CECT, and CEMR enabled determination of left PV patency in 33.3%, 80.0%, and 75.0% of studies, respectively. Ultrasound with Doppler was diagnostic in 77.8% of the examinations. Trans-splenic portography was unable to adequately assess the status of the left PV in any studies (0%) and transarterial portography definitively answered this question in only 41.6% of studies. Transhepatic portography provided the greatest diagnostic capability for the left PV among the planar angiographic techniques, at 90.9%. CT arterial portography reliably enabled determination of left PV patency in 100% of the studies. For determining left PV patency, the differences between modalities were statistically significant (*P* = .039).

**Table 1:** Diagnostic capability by modality.

	Diagnostic capability		
Modality	Left PV patency or occlusion	SMV patency or occlusion	PV contiguity or non-contiguity
Single phase CECT	1/3 (33.3)	3/3 (100)	1/3 (33.3)
Multi-phase CECT	8/10 (80.0)	10/10 (100)	4/10 (40.0)
Multi-phase CEMR	3/4 (75.0)	2/4 (50.0)	1/4 (25.0)
Ultrasound	7/9 (77.8)	NA	1/9 (11.1)
Trans-splenic portography	0/2 (0)	NA	0/2 (0)
Transhepatic portography	10/11 (90.9)	NA	9/11 (81.8)
Transarterial portography	5/12 (41.6)	9/12 (75.0)	1/12 (8.3)
CTAP	6/6 (100)	6/6 (100)	6/6 (100)

*Note:* Values are number of studies. Numbers in parentheses are percentages.

With respect to assessment of SMV patency or occlusion, single-phase, multi-phase CECT, and CTAP performed equally well, all enabling confident diagnosis in 100% of the examinations. Conversely, confident diagnosis regarding the status of the SMV could be made in only 50.0% of CEMR examinations and 75.0% of transarterial portograms. The differences between all modalities regarding SMV patency were not statistically significant (*P* = .302).

The ability to demonstrate contiguity versus non-contiguity of the intrahepatic right and left PVs varied widely. Adequate demonstration of intrahepatic portal venous anatomy was achieved in 33.3% of single-phase and 40.0% of multi-phase CECT examinations. CEMR, ultrasound with Doppler, and transarterial portography were valuable in only one examination each (25.0%, 11.1%, and 8.3%, respectively). Trans-splenic portography failed to confidently reveal the presence or absence communication between the intrahepatic PVs in any of the studies. Transhepatic portography elucidated relevant intrahepatic portal venous anatomy in 81.8% of the studies. CT arterial portography reliably provided diagnostic quality images of the intrahepatic PVs, allowing for determination of PV contiguity in 100% of studies. For determining PV contiguity, the difference between modalities was statistically significant (*P*<.001).

Subgroup analysis of the patients that underwent CTAP showed that all had undergone other types of abdominal imaging. For five out of six patients, CTAP enabled confident determination of PV contiguity where the other imaging modalities were non-diagnostic. In the remaining patient, transarterial portography was also diagnostic. CTAP also allowed for confident determination of left PV and SMV patency or occlusion for all patients; however, the other imaging modalities obtained were also able to characterize these structures.

Nine patients ultimately received a Rex shunt; there were no noted discrepancies between findings at surgery and the preoperative imaging findings. However, not all structures assessed with imaging were evaluated during surgery. In particular, PV contiguity was not assessed during surgery. Five underwent distal splenorenal shunting per the preferences of the liver surgeon. Two patients received a liver transplant. Four patients have not undergone definitive surgical treatment.

## Discussion

Patients with long standing EPVT often have massive cavernous collateral vessels at the porta hepatis which obscure the dominant left and right PVs and pose a diagnostic challenge. Cross-sectional imaging of the portal venous system with contrast-enhanced CT (CECT) can be limited by low contrast resolution. CEMR affords high contrast resolution of the portal venous system, but the lengthy sequences required for adequate spatial resolution are prone to motion artifact and this imaging modality frequently requires general anesthesia in the pediatric population. Ultrasound with Doppler has a small field of view which makes differentiation of cavernous collaterals from intrahepatic PVs very difficult. Ultrasound also does not allow for assessment of the SMV. The two-dimensional projectional nature of conventional angiography limits characterization of complex portal venous anatomy. Transhepatic and trans-splenic portography also carry with them increased risk of complications associated with puncture of highly vascular organs. Transarterial portography is relatively less risky and more technically straight-forward.

**Figure 1:**
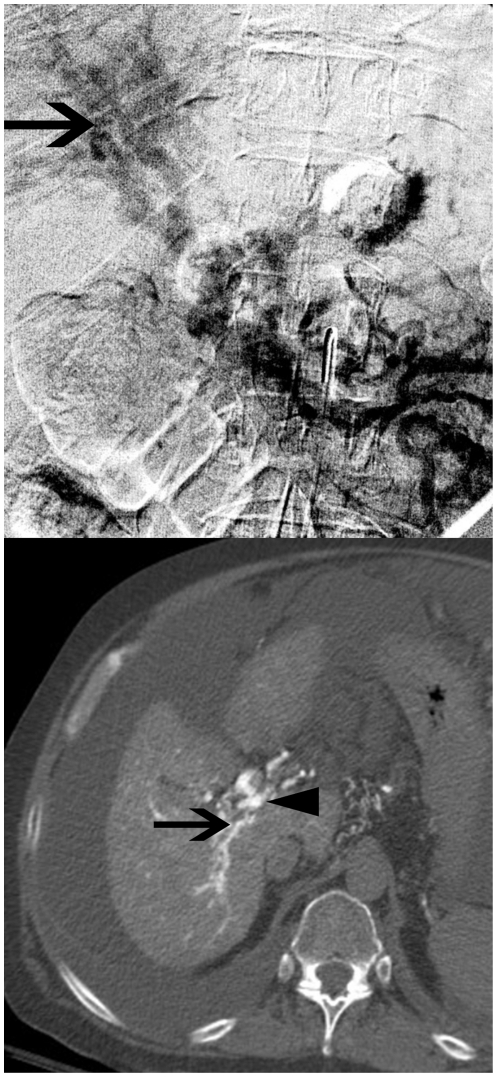
Images from a 59-year-old male with EPVT undergoing evaluation for possible Rex shunt. a) Digital subtraction angiographic image from transarterial portography shows patent intrahepatic PVs (arrow), but the presence or absence of a connection between the left and right systems cannot be determined. b) Transaxial image from CTAP demonstrates a complex network of intrahepatic PVs and connection between the left (arrowhead) and right (arrow) PVs.

**Figure 2:**
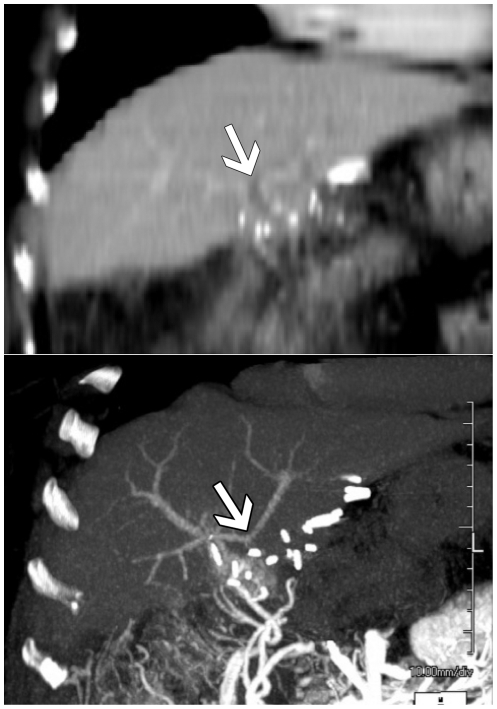
64-year old female status post liver transplant with EPVT undergoing evaluation for possible Rex shunt. a) Coronal maximum intensity projection image from a multi-phase CECT does not clearly show the connection between the left and right PVs (arrow). b) Coronal maximum intensity projection image from CTAP clearly demonstrates contiguity between the intrahepatic left and right PVs (arrow).

CTAP allows for cross-sectional interrogation, high contrast-to-noise ratio, high spatial resolution and a large field of view. These attributes facilitate characterization of the portal venous system despite the presence of a complex network of collateral vessels (Figures 1 and 2). These advantages of CTAP over the other modalities are reflected in the results of this study, with CTAP allowing for confident assessment of the intrahepatic PV anatomy and left PV patency or occlusion in all examinations. Furthermore, all but one of these patients had undergone prior non-diagnostic examinations, suggesting that these patients had relatively challenging venous anatomy.

A disadvantage of CTAP includes the requirement for angiographic catheter placement. It is important to note that pediatric patients constitute a substantial portion of patients evaluated for Rex shunt placement. While complications of femoral artery catheterization are rare in all age groups, when they occur in very young children, the outcomes are poor [8]. CTAP also requires coordination to smoothly transition patients between the angiography suite and the CT scanner. However, should combined cone-beam CT/angiography systems become more widely available, this issue will become obsolete.

Little has been written on the appropriate imaging algorithm for patients undergoing evaluation for Rex shunt. Bambini et al. reported a case series of five patients who underwent successful Rex shunt placement [2]. Preoperative imaging assessment of the intrahepatic PVs included ultrasound, transhepatic portography, and transarterial portography; these examinations were reported to be non-diagnostic or inaccurate in the majority of cases. The authors concluded that available imaging techniques were unreliable for determining adequacy of the intrahepatic PVs and recommended direct visualization at surgery. CTAP was not used as an imaging modality in this cohort of patients.

At our institution, multiple different imaging modalities are used; the choice of modalities is variable and has evolved over time. In the beginning of the study period, patients tended to undergo transhepatic and trans-splenic portography for their initial imaging. When these studies were non-diagnostic, cross-sectional imaging with CECT or CEMR was obtained. Later in the study period, preoperative imaging evaluation shifted, reflecting a trend towards CECT and ultrasound as the preferred initial imaging modalities. When these studies were non-diagnostic, transhepatic portography or CTAP were performed to further characterize the portal venous system.

Given the excellent diagnostic capability of CTAP for determining the key criteria for surgical candidacy, this modality should be considered as a first or second line examination for the preoperative imaging evaluation of patients with EPVT. A full cost analysis for different ordering patterns is beyond the scope of this study. A reasonable algorithm may begin with a CECT. If the relevant vessels are well-assessed, no additional imaging would be indicated. This spares the patient from undergoing any invasive imaging study. An additional advantage of starting with CECT is that those patients who are not candidates for Rex shunt placement may be candidates for distal splenorenal shunting and CECT provides information about the splenic and renal veins. If the surgical criteria are not adequately assessed with CECT, CTAP could subsequently be performed.

This study did not attempt to compare the accuracy of the different imaging modalities. The majority of patient did not undergo Rex shunt surgery and even those that did undergo surgery did not have surgical confirmation of PV contiguity. Because no true gold standard existed, we were limited to the assessment of diagnostic capacity, not diagnostic performance. This study was also limited by the non-standardized imaging protocols between modalities. For example, comparison between CECT images acquired with 2.5 mm collimation and CTAP images with 1.25 mm collimation could be affected by the slice thickness. Nonetheless, the examinations were performed in accordance with standard clinical protocols employed at our institution. As this was a retrospective study of examinations ordered based on referring surgeons' preferences, we could not perform a within-patient analysis between the different modalities.

In summary, our study suggests that, of the imaging modalities that have been employed for Rex shunt preoperative planning, CTAP most reliably allows for confident assessment of left PV patency, SMV patency, and left and right PV contiguity in a single examination.
